# Reversible ADP-ribosylation of RNA

**DOI:** 10.1093/nar/gkz305

**Published:** 2019-04-26

**Authors:** Deeksha Munnur, Edward Bartlett, Petra Mikolčević, Ilsa T Kirby, Johannes Gregor Matthias Rack, Andreja Mikoč, Michael S Cohen, Ivan Ahel

**Affiliations:** 1 Sir William Dunn School of Pathology, University of Oxford, South Parks Road, Oxford OX1 3RE, UK; 2 Division of Molecular Biology, Ruđer Bošković Institute, Zagreb, Croatia; 3 Program in Chemical Biology, Department of Physiology and Pharmacology, Oregon Health and Science University, Portland, OR 97239, USA

## Abstract

ADP-ribosylation is a reversible chemical modification catalysed by ADP-ribosyltransferases such as PARPs that utilize nicotinamide adenine dinucleotide (NAD^+^) as a cofactor to transfer monomer or polymers of ADP-ribose nucleotide onto macromolecular targets such as proteins and DNA. ADP-ribosylation plays an important role in several biological processes such as DNA repair, transcription, chromatin remodelling, host-virus interactions, cellular stress response and many more. Using biochemical methods we identify RNA as a novel target of reversible mono-ADP-ribosylation. We demonstrate that the human PARPs - PARP10, PARP11 and PARP15 as well as a highly diverged PARP homologue TRPT1, ADP-ribosylate phosphorylated ends of RNA. We further reveal that ADP-ribosylation of RNA mediated by PARP10 and TRPT1 can be efficiently reversed by several cellular ADP-ribosylhydrolases (PARG, TARG1, MACROD1, MACROD2 and ARH3), as well as by MACROD-like hydrolases from VEEV and SARS viruses. Finally, we show that TRPT1 and MACROD homologues in bacteria possess activities equivalent to the human proteins. Our data suggest that RNA ADP-ribosylation may represent a widespread and physiologically relevant form of reversible ADP-ribosylation signalling.

## INTRODUCTION

Adenosine diphosphate (ADP)-ribosylation is a covalent modification in which the ADP-ribose (ADPr) group from nicotinamide adenine dinucleotide (NAD^+^) is transferred to diverse target molecules: proteins, nucleic acids and small molecules such as phosphate or acetate (1). This modification changes physical and chemical properties or localization of target molecules and regulates many important cellular processes in both prokaryotes and eukaryotes (2).

ADP-ribosylation was first described as a mechanism of pathogenicity used by pathogenic bacterial exotoxins that irreversibly modify crucial host cell proteins (3). Two divergent bacterial toxins, diphtheria and cholera toxin are founders of two major ADP-ribosyl transferase (ART) groups (4,5). Poly(ADP-ribose) polymerases (PARPs), the best studied and largest ART subgroup, belong to diphtheria toxin-like ADP-ribosyl transferases. PARPs are present in all eukaryotes (except yeast) and sporadically in bacteria; they regulate important cellular processes such as DNA damage repair, transcription, protein degradation, cell-cycle progression, host-virus interaction, cell division, ageing, cell death and bacterial metabolism (6–9). The human genome encodes for seventeen PARPs with different domain architecture and functions (2). tRNA 2′-phosphotransferase 1 (TRPT1/Tpt1/KptA) is sometimes referred to as the eighteenth PARP family member (4). Several PARP family members (PARP1, PARP2 and tankyrases) synthesize long chains of poly-ADPr, while the other PARP family members transfer a single ADPr group on targets (such as PARP3 and PARP16) (7).

ADP-ribosylation is a dynamic chemical modification that is regulated both at the level of addition and the removal of ADPr groups. PARPs have been shown to target mostly Glu/Asp or Ser residues (8–12). Poly-ADP-ribosylation can be removed by the action of two divergent enzymes, poly(ADP-ribose) glycohydrolase (PARG) and ADP-ribosylhydrolase 3 (ARH3) (13,14). PARG is unable to remove the last ADPr group attached to target proteins (13) while ARH3 is the only hydrolase that can completely remove both poly- and mono-ADPr signal from serine residue (15), a modification catalysed by PARP1/HPF1 and PARP2/HPF1 complexes (11,16). Terminal ADPr linked to Glu/Asp is removed by macrodomain containing enzymes namely terminal ADPr glycohydrolase 1 (TARG1/OARD1), MACROD1 and MACROD2 (17–20). Enzymes with phosphodiesterase activity, NUDT16 (nucleoside diphosphate-linked moiety X-type motif 16) and ENPP1 (ectonucleotide pyrophosphatase/phosphodiesterase 1), can cleave pyrophosphate from both poly- and mono-ADPr modified targets leaving phosphoribose tags on the proteins (21,22).

Although ADP-ribosylation has historically been considered to mostly target proteins, there has been increasing evidence that DNA can also be a target for ADP-ribosylation. The first enzymes reported to ADP-ribosylate DNA were pierisins, toxins expressed by cabbage butterfly and related species. Piersins irreversibly ADP-ribosylate DNA on guanines (23,24). More recently, it was discovered that the bacterial toxin-antitoxin system DarT-DarG mediates reversible DNA ADP-ribosylation on thymidine residues in single-stranded DNA (in a sequence specific manner) (25). Furthermore, it was shown that DNA repair PARPs (PARP1, PARP2 and PARP3) can modify DNA on phosphates at DNA breaks (26–29). The ADPr groups on phosphates at DNA breaks are efficiently removed by several cellular hydrolases, most notably by PARG, MACROD1/2, TARG1 and ARH3 (26,27,30).

The pool of cellular substrates for ADP-ribosylation has continued to expand and it was recently shown that a PARP-like proteins TRPT1/Tpt1/KptA from bacteria and fungi can ADP-ribosylate RNA and DNA ends (31).

In this paper, we reveal that ADP-ribosylation of RNA at the terminal phosphate is more widespread than initially thought. We demonstrate that homologues of TRPT1 in higher organisms as well as human PARP10, PARP11 and PARP15 can ADP-ribosylate phosphorylated ends of RNA. We also show that RNA ADP-ribosylation is a reversible process that can be accomplished by several human hydrolases as well as by some viral and bacterial macrodomains. Thus, this study provides the first evidence of reversible ADP-ribosylation of RNA.

## MATERIALS AND METHODS

### Plasmid and protein purification

Plasmids expressing full length (FL) PARP3 was cloned into pDEST17 vector with His tag. cDNA encoding the human PARP4 catalytic and BRCT domains (1-572aa) were obtained using gBlock gene fragments and cloned into a pET-His-SUMO-TEV using ligation independent cloning. PARP5bcat (Tankyrase2 catalytic domain) was cloned into pET-His6 vector. PARP10 catalytic domain (818-1025aa) WT and G888W mutant genes were cloned into pGEX-4T1 vector with GST tag. PARP10 FL and PARP16 FL genes were cloned into pET-His6-SUMO-TEV vector. PARP11 catalytic domain (128–338aa) was cloned in pET28a vector. The catalytic domain of human PARP12 (489–684aa) was PCR-amplified from the cDNA library using primers with non-complementary restriction enzyme sites located at the 5′ (EcoRI) and 3′ (XhoI) ends. The amplified product was cloned into pET-28b+ (Novagen). PARP13 catalytic domain (716–902aa) was PCR amplified and cloned into pET28a vector using BamHI and XhoI cloning sites. PARP14 wwe and catalytic domain (1459–1801aa) and PARP15 catalytic domain (481-678aa) were cloned into pNic-Bsa4-6xHis vector. TRPT1 gene (Uniprot Q86TN4) was codon optimized and synthesized with His tag from Invitrogen GeneArt Gene synthesis and then further cloned into pET28a vector using NcoI and XhoI restriction enzyme cloning. Point mutants of TRPT1 were prepared by using Agilent Quick Change Lighting Site Directed Mutagenesis kit. *Streptomyces coelicolor* KptA homologue (SCO3953) was cloned from *S. coelicolor* genomic DNA into pET15b.

PARP3 FL was purified as mentioned earlier (27). PARP4, Tankyrase2 cat (32), PARP12 cat and PARP16 FL plasmids were transformed into Escherichia coli BL21 (DE3) competent cells (Millipore) and grown on LB agar plates with Kanamycin (50 mg/ml) and Chloramphenicol (34 mg/ml) overnight at 37°C. A swath of cells were inoculated into a 50 ml starter culture of LB media with Kanamycin and Chloramphenicol at 225 rpm, 37°C overnight. For each protein of interest 1 L of terrific broth (TB) media (12 g Bacto Tryptone, 24 g yeast extract, 0.4% glycerol, 17 mM KH_2_PO_4_, 72 mM K_2_HPO_4_, 1% glucose, 50 μg/ml Kanamycin, 34 μg/ml Chloramphenicol) was inoculated with the starter culture and grown to 0.8–1.0 OD_600_ at 37°C, 225 rpm. IPTG (Sigma-Aldrich) was added to 0.4 mM to induce protein expression for 18–24 h at 16°C, 225 rpm. Cells were harvested by centrifugation, resuspended in lysis buffer (20 mM HEPES, pH 7.5, 1 mM β-mercaptoethanol, 1 mM benzamidine, 0.2% NP-40, 0.2% Tween-20, 500 mM NaCl, 1 mM phenylmethylsulfonyl fluoride (PMSF), 8.3 mg/l DNAse I (Roche)) and lysed by sonication at 0°C (Branson sonifier 450). Lysates were incubated with pre-washed Ni-NTA agarose resin (50% slurry, Qiagen) with end-over-end rotation at 4°C for 1 h. Following extensive washing with buffer B1+20 (20 mM HEPES, pH 7.5, 1 mM β-Me, 1 mM PMSF, 1 mM benzamidine, 500 mM NaCl, 20 mM imidazole) protein was eluted in four fractions of B1 containing 100–400 mM imidazole. Fractions containing protein were collected and dialysed against 50 mM Tris–HCl, pH 7.5, 0.1 mM EDTA, 1 mM β-Me, 0.4 M NaCl at 4°C.

PARP10 catalytic domain (WT and G888W mutant) were purified as mentioned earlier (22). In short, GST-tagged PARP10 was transformed into Rosetta DE3 competent cells and grown in LB media supplemented with Ampicillin and Chloramphenicol. Cultures were induced with 0.5  mM IPTG at 0.8–1.0 OD_600_ and grown overnight at 16 °C. Following centrifugation, PARP10 cat bacterial cell pellet was resuspended in PBS buffer supplemented with BugBuster protein extraction reagent, Benzonase, 10% glycerol, 1 mM DTT and Complete Protease inhibitor cocktail and allowed to lyse by incubation at 4°C for 1 h. Lysate was further centrifuged and cleared lysate was applied to glutathione sepharose beads for 1 h at 4°C. GST-tagged PARP10 was eluted using lysis buffer supplemented with 20 mM reduced glutathione. Eluted protein was further dialysed against 25 mM Tris–HCl pH 7.5, 150 mM NaCl, 10% glycerol and 1 mM DTT. PARP10 cat WT and G888W mutant were further purified on Superdex 200 column.

PARP10 FL was transformed into Rosetta DE3 competent cells and grown in 6 l 2× YT media supplemented with Kanamycin. Cultures were induced with 0.5 mM IPTG at 0.6–0.8 OD_600_ and grown overnight at 18°C. Bacterial pellet was resuspended in lysis buffer (20 mM HEPES pH 8, 500 mM NaCl, 10 mM imidazole and 0.5 mM TCEP). PARP10 FL was purified via three-step purification process involving Nickel column purification, heparin column and gel filtration column. Cells were lysed by addition of BugBuster, protease inhibitor cocktail, benzonase and lysozyme and allowed to lyse for 1 h at 4°C. Cleared lysate was incubated with pre-washed Ni-NTA agarose resin (50% slurry, Qiagen) with end-over-end rotation at 4°C for 1 h. Beads were then further washed with high salt buffer (20 mM HEPES pH 8, 1 M NaCl, 10 mM imidazole and 0.5 mM TCEP) followed by gradient elution over 10 mM–1 M imidazole. Eluted protein was assessed by SDS-PAGE gel and further dialysed against 25 mM Tris pH 7.5, 100 mM NaCl, 1 mM EDTA and 0.1 mM TCEP. Dialysed protein sample was further diluted using no salt buffer (25 mM Tris pH 7.5, 1 mM EDTA and 0.1 mM TCEP) to get the salt concentration to 30 mM NaCl and was applied onto Heparin column to remove any nucleic acid contamination. Small fraction of protein bound onto Heparin column while most ran out as flow through in the condition tested. The Heparin column bound protein was eluted with gradient of 30 mM–1 M NaCl concentration. At this stage, the purity of eluted protein was tested by SDS-PAGE gel. Protein fractions were concentrated and further subjected to size exclusion chromatography using Superdex 200 column.

PARP13 cat (716-902aa) and PARP14 wwe and cat (1459–1801aa) plasmids were transformed in Rosetta DE3 competent cells and grown in 2× YT media supplemented with Kanamycin. Induction was carried out at 0.6–0.8 OD_600_ using 0.5 mM IPTG and cells were allowed to grow overnight at 18°C. Bacterial pellet was lysed in lysis buffer (20 mM HEPES pH 8, 500 mM NaCl, 10 mM imidazole and 0.5 mM TCEP) supplemented with BugBuster, protease inhibitor cocktail, benzonase and lysozyme. Cleared lysate was then bound to pre-washed Ni-NTA agarose resin followed by washes with lysis buffer. Proteins were eluted using elution buffer (20 mM HEPES pH 8, 500 mM NaCl and 0.5 mM TCEP) with an incremental gradient of 10–500 mM imidazole. Proteins purity was assessed by SDS-PAGE gel. PARP13 protein was dialysed overnight against 25 mM Tris pH 7.5, 100 mM NaCl, 1 mM EDTA and 0.1 mM TCEP buffer. PARP14 protein was dialysed overnight against 20 mM HEPES pH 7.5, 300 mM NaCl and 0.5 mM TCEP and further subjected to Superdex 75 column for size exclusion chromatography. The catalytic domain of PARP15 (481–678aa) was purified as mentioned earlier (33).

TRPT1 was purified as described earlier (34), in short TRPT1 plasmid was transformed into Rosetta DE3 competent cells and grown in LB media supplemented with Kanamycin. Cultures were induced with 0.2–0.5 mM IPTG at 0.8 OD_600_ and grown overnight at 16 °C. Following centrifugation, TRPT1 bacterial pellet (WT or mutants) was resuspended in lysis buffer (50 mM Tris–HCl pH 7.5, 150 mM NaCl, 10% glycerol and 10 mM imidazole) supplemented with BugBuster, Benzonase, 0.5 mM TCEP and Complete protease inhibitor cocktail and lysed by mixing for 1 h at 4°C. Lysate was centrifuged at 17 000 rpm for 50 min and the cleared lysate was incubated with prewashed nickel NTA agarose beads for 1 h at 4°C. His-tagged TRPT1 protein was eluted using elution buffer (50 mM Tris–HCl pH 8, 0.1 M NaCl, 10% glycerol) with an incremental gradient of 10–500 mM imidazole. Eluted TRPT1 protein was dialysed overnight against column buffer (50 mM Tris–HCl pH 8, 50 mM NaCl, 2 mM DTT and 10% glycerol). TRPT1 WT protein was further purified by size exclusion chromatography using Superdex 75 column.

*Streptomyces coelicolor* KptA homologue (SCO3953) gene was expressed in *Escherichia coli* BL21(DE3) cells and were grown for 3 h at 30°C with 0.8 mM IPTG added at 0.8 OD_600_. Recombinant protein was purified using TALON affinity resin according to standard procedure. *Streptomyces coelicolor* MacroD homologue (SCO6450) was obtained as described earlier (30).

Proteins listed below were gifts from other members of the lab. *Mycobacterium tuberculosis (Mtb)* DarG-macro was cloned with 155 N-terminal amino acids as described earlier (25). Catalytic domains of PARP11 (128-338aa) (35), MACROD1 (30,36), MACROD2 (19), TARG1 (17), PARG (37), NUDT16 (21) and ARH 1‐3 (15) were purified as described earlier. Viral macrodomain-containing hydrolases from VEEV (38) and SARS Coronavirus (39) were prepared as described earlier.

### Oligonucleotide

Single stranded (ss) RNA and DNA oligos used in this study were commercially ordered from Sigma-Aldrich and Invitrogen, respectively, and are listed in Table 1. Oligonucleotides were diluted to 100 μM stock solution in 20 mM HEPES–KOH (pH 7.6) and 50 mM KCl buffer. Double stranded (ds) DNA was prepared by annealing complementary strands of DNA (ssDNA oligo with RexT) at 95°C for 5 min and then allowed to gradually cool down to room temperature.

**Table 1.** Sequence of oligonucleotides used in this study

Name	Sequence (5′→3′)
5P ssRNA	[Phos] GUGGCGCGGAGACUUAGAGAA
3P ssRNA	GUGGCGCGGAGACUUAGAGAA [Phos]
noP ssRNA	GUGGCGCGGAGACUUAGAGAA
3P ssRNA 5Cy3	[Cy3] GUGGCGCGGAGACUUAGAGAA [Phos]
noP ssRNA 5Cy3	[Cy3] GUGGCGCGGAGACUUAGAGAA
5P ssRNA 3Cy3	[Phos] GUGGCGCGGAGACUUAGAGAA [Cy3]
noP ssRNA 3Cy3	GUGGCGCGGAGACUUAGAGAA [Cy3]
5P ssDNA	[Phos] GTGGCGCGGAGACTTAGAGAA
3P ssDNA	GTGGCGCGGAGACTTAGAGAA [Phos]
noP ssDNA	GTGGCGCGGAGACTTAGAGAA
RexT DNA	GGAATTCCCCGCGCCAAATTTCTCTAAGTCTCCGCGCCAC
7mer	[Phos] GUGGCGC [Cy3]
12mer	[Phos] GUGGCGCGGAGA [Cy3]

10 pmol noP ssRNA (with or without Cyanine3 tag at 3′ end) was radioactively labelled at 5′ end using T4 polynucleotide kinase 3′ phosphatase minus (NEB) in presence of γ^32^P ATP (Perkin Elmer) and heated at 37°C for 30 min followed by heat inactivation at 65°C for 20 min. Radiolabelled oligo was further desalted on G25 column to remove any unincorporated ATP. This radiolabelled oligo was used as size marker as indicated in figures.

### ADP-ribosylation assay

ADP‐ribosylation assays with RNA were performed as described previously for DNA ADP-ribosylation (27). All buffers were made in DNase/RNase free water and filter sterilized prior to use. In short, 10 μl reaction mix was prepared in buffer containing 20 mm HEPES–KOH (pH 7.6), 50 mm KCl, 5 mm MgCl_2_ and 1 mm DTT. RNA substrate (10 μm) was added along with 2 μm protein, 50 μm NAD^+^ (Trevigen) and 50 kBq ^32^P labelled NAD^+^ (PerkinElmer) per reaction. Protein and NAD^+^ concentrations were used as mentioned above unless stated otherwise. Reactions were incubated at 37°C for 30 min and stopped by addition of 50 ng/μl Proteinase K and 0.15% SDS and heating the reaction at 50°C for 30 min, unless stated otherwise. Reactions that were treated with Benzonase or calf intestinal phosphatase (CIP) were heated at 37°C for 30 min. Samples were further heated at 95°C for 3 min with 2× TBE urea sample buffer (8 m urea, 20 μm EDTA pH 8.0, 2 μm Tris pH 7.5 and bromophenol blue). The samples were loaded on a pre-run denaturing urea PAGE gel made of 20% (w/v) polyacrylamide, 8 m urea and 1× TBE. The gel was run at 7 W/gel in 0.5× TBE buffer. The gel was dried under vacuum and visualized by autoradiography.

Non-radioactive RNA ADP-ribosylation assay was performed using Cyanine3 labelled RNA essentially similar to radioactive assay with exception of using 1 μM labelled RNA oligo and 500 μM NAD^+^. The gel was visualized using Molecular Imager PharosFX systems using laser excitation for Cyanine3 fluorophore at 532 nM wavelength. All ADP-ribosylation assays were individually repeated three times.

To study the effect of time kinetics on PARP10 cat and TRPT1 mediated RNA ADP-ribosylation, reaction samples were prepared as mentioned earlier. Aliquots were taken out at different time points (3, 10 and 30 min). The 0 min time was done by placing the reaction on ice. Reactions were stopped by addition of 2× TBE urea sample buffer.

To study the effect of NAD^+^ concentration dependence of PARP10 cat and TRPT1 the assay was performed as described earlier with different concentrations (0–1500 μM) of NAD^+^ in reaction. NAD^+^ concentration dependence study was performed in non-radioactive setup using Cyanine3 labelled RNA.

RNA ADP-ribosylation reaction studying the effect of adenosine mono-phosphate (AMP) or 5′-phosphoadenosine 3′ phosphate (PAP) on PARP10 mediated RNA modification was performed by supplementing the reaction with 0, 50 or 250 μM concentration of AMP or PAP.

### ADP-ribosylhydrolase assay

PARP10 catalysed RNA ADP-ribosylation reactions were stopped by addition of PARP inhibitor, 3-aminobenzamine (3ABA) before treating with hydrolases. 2 μm hydrolase enzymes were added per reaction and heated at 30°C for 30 min. Reactions containing NUDT16 were supplemented with 15 mm MgCl_2_ (21).

## RESULTS

### PARP10 can ADP-ribosylate phosphorylated RNA ends

**Figure 1.**
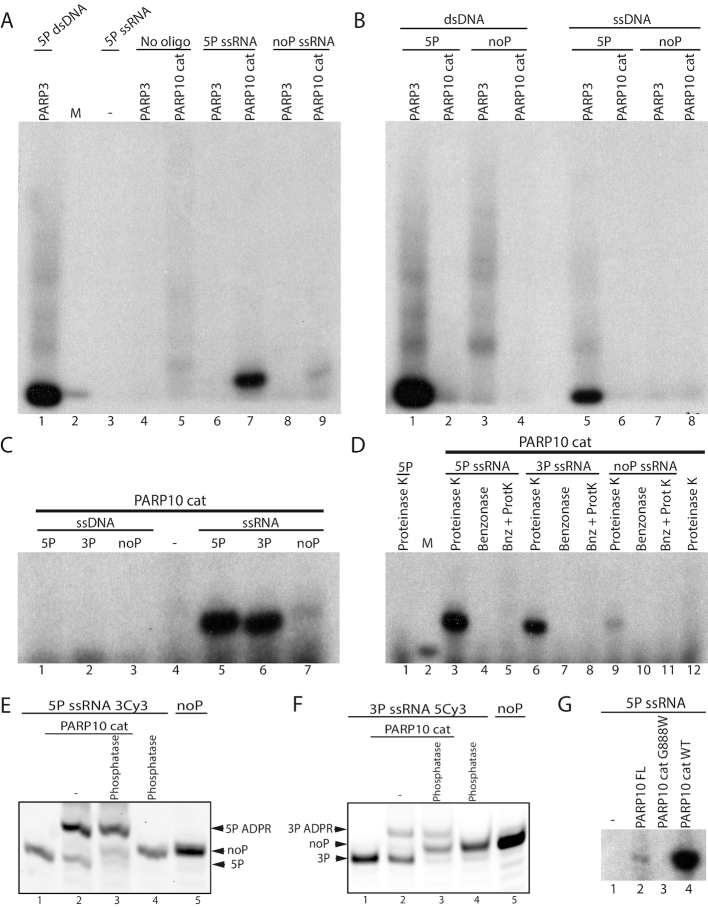
ADP-ribosylation of RNA mediated by PARP10. (**A**) ADP-ribosylation of phosphorylated or non-phosphorylated single-stranded (ss) RNA substrate in the presence of PARP3 or PARP10 cat. ^32^P labelled ssRNA was used as a marker in lane 2 and PARP3 mediated DNA ADP-ribosylation was used as a positive control for nucleic acid ADP-ribosylation in lane 1. (**B**) ADP-ribosylation by PARP3 and PARP10 of recessed double-stranded (ds) or ssDNA. (**C**) Specific ADP-ribosylation of ss DNA or RNA with or without phosphate group at 5′ or 3′ end of the oligo by PARP10. (**D**) PARP10 catalysed RNA ADP-ribosylated substrate treated with: Proteinase K & SDS, Benzonase alone or Benzonase followed by Proteinase K (Bnz + ProtK). (**E**) ssRNA with phosphate group at 5′ end and labelled with Cyanine3 tag at 3′ end (5P ssRNA 3Cy3) and (**F**) ssRNA with phosphate group at 3′ end and labelled with Cyanine3 tag at 5′ end (3P ssRNA 5Cy3) modified in the presence of PARP10 and further treated with CIP. Gels in (E) and (F) are visualized using Cyanine3 label and PharosFX imager. (**G**) RNA ADP-ribosylation catalysed by PARP10 full length and PARP10 catalytic domain WT and G888W mutant.

In recent years there has been increasing evidence of DNA as a new target for reversible ADP-ribosylation. We wanted to investigate whether RNA could also be similarly ADP-ribosylated by any known ARTs. We decided to initially test PARP3 as it was recently demonstrated that this protein has robust ART activity on DNA (27–29). In addition, we focused on another member of the PARP family, PARP10, which contains a RNA-recognition motif (RRM domain) (40). Purified PARP3 and PARP10 (catalytic domain) were first tested with a 21 nucleotide single-stranded RNA (ssRNA) oligo with or without a phosphate group at the 5′ end in the presence of ^32^P labelled NAD^+^ as an ADPr donor. Double-stranded DNA was used as a positive control (Figure 1A, lane 1) for ADP-ribosylation on DNA by PARP3 (27). Strikingly, PARP10 substantially modified the 5′ phosphorylated ssRNA oligo (Figure 1A, lane 7) reducing its mobility compared to the phosphorylated oligo labelled on its 5′ phosphate using ^32^P labelled gamma-ATP (Figure 1A, lane 2). PARP10 was not able to ADP-ribosylate ssRNA without a 5′ phosphate group (Figure 1A, lane 7 compared to lane 9), suggesting that RNA ADP-ribosylation by PARP10 occurs on the 5′ phosphate. Importantly, PARP3 could only ADP-ribosylate DNA ends and did not have any activity on RNA oligos, while PARP10 specifically modified phosphorylated ssRNA oligo in the conditions tested (Figure 1A and B). We further demonstrate RNA ADP-ribosylation activity of PARP10 is time and NAD^+^ concentration dependent (Supplementary Figure S1A and B) but independent of MgCl_2_ presence (Supplementary Figure S1C, lanes 1 and 2). To further ascertain the specificity of ADP-ribosylation by PARP10 on DNA and/or RNA ends we tested both single-stranded oligos with or without a phosphorylated moiety at either 5′ or 3′ end. PARP10 modified ssRNA oligos phosphorylated at either end but did not show activity on ssDNA oligo irrespective of the terminal phosphorylation state (Figure 1C, lanes 5 and 6). The reaction product catalysed by PARP10 in presence of phosphorylated ssRNA was stable against Proteinase K treatment (Figure 1D, lanes 3 and 6) but not against treatment with Benzonase (Figure 1D, lanes 4 and 7) confirming that the modification was on nucleic acid and not on protein. Next we wanted to assess the specificity of PARP10 for modification of the phosphate groups on RNA. For this, we analysed RNA ADP-ribosylation catalysed by PARP10 catalytic domain in the excess presence of adenosine mono-phosphate (AMP) or 5′-phosphoadenosine 3′ phosphate (PAP) as potential competitors and we observed no significant change in RNA modification in presence of nucleotide analogue in excess (Supplementary Figure S1D).

To further demonstrate that ADP-ribosylation of RNA by PARP10 occurs on terminal phosphates we designed 5′ and 3′ phosphorylated ssRNAs with the Cyanine3 (Cy3) label at the opposite end to the phosphorylation for more sensitive detection. We also produced non-phosphorylated versions of these oligos as additional controls. The phosphorylated oligos 5P ssRNA 3Cy3 and 3P ssRNA 5Cy3 treated with PARP10 produced a slower migrating ADP-ribosylated product (Figure 1E and F, lane 2). When the reaction was further treated with calf intestinal phosphatase (CIP, phosphatase), the slower migrating ADP-ribosylated band remains intact while the lower unmodified band shifts upwards due to the removal of a charged phosphate group by CIP phosphatase treatment (Figure 1E and F, lane 3) and now migrates the same as non-phosphorylated oligo control (Figure 1E and F, lane 5). When the phosphorylated oligos 5P ssRNA 3Cy3 and 3P ssRNA 5Cy3 (Figure 1E and F, lane 1) are treated directly with CIP in absence of PARP10, dephosphorylation of oligo is observed which is confirmed by similar migrating pattern as the non-phosphorylated oligo (Figure 1E and F, lanes 4 and 5). PARP10 modified 5′ phosphorylated end more efficiently than 3′ phosphorylated RNA end. These results show that ADP-ribosylation of RNA by PARP10 occurs on the phosphate group thereby protecting the phosphate group from the dephosphorylation activity of CIP. Since the catalytic domain of PARP10 lacks two-thirds of the protein including the RRM we wanted to assess the activity of the FL PARP10 on RNA substrate. We expressed and purified full length PARP10 and tested it for RNA modification activity. We observed PARP10 full length can also ADP-ribosylate phosphorylated RNA ends (Figure 1G, lane 2), however, in comparison to WT PARP10 catalytic domain the activity was much weaker (Figure 1G, lane 4). A possible explanation for this finding may be that the isolated FL PARP10 exists in an autoinhibited state, due to the inhibitory function of another domain within the protein. Such autoinhibitory property has been already well characterized for PARP1 (41). We observed no RNA modification by previously characterized catalytic mutant of PARP10 G888W (Figure 1G, lane 3).

### ADP-ribosylation of RNA ends by PARP10 is a reversible process

**Figure 2.**
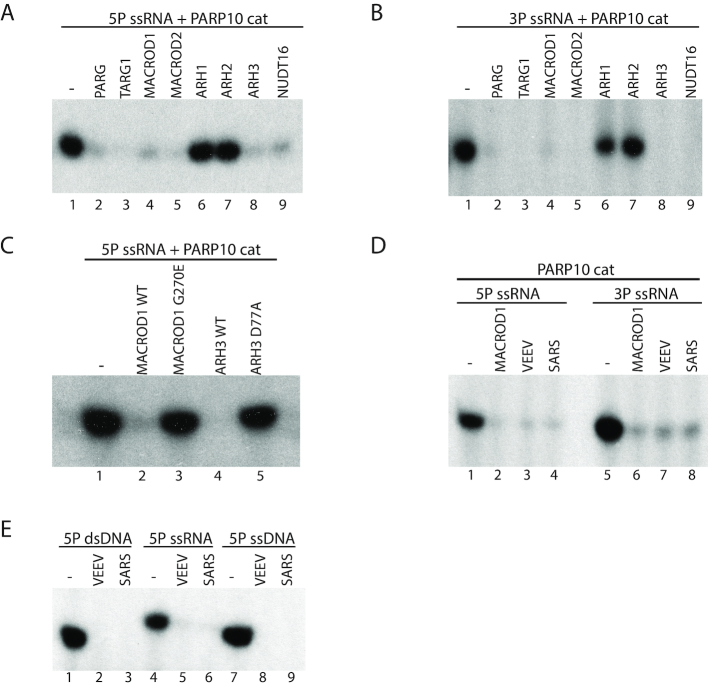
Reversal of PARP10 mediated RNA ADP-ribosylation by human hydrolases at (**A**) 5′ phosphorylated and (**B**) 3′ phosphorylated end of ssRNA. (**C**) ADP-ribosylhydrolase activity of WT or mutant MACROD1 and ARH3 on 5P ADP-ribosylated PARP10 substrate. (**D**) PARP10 mediated RNA ADP-ribosylation at 5′ and 3′ ends can be reversed by macrodomain proteins from viral origin such as VEEV and SARS using human MACROD1 as a positive control for ADP-ribosylhydrolase activity. (**E**) Viral macrodomains of VEEV and SARS can also reverse dsDNA, ssDNA and ssRNA ADP-ribosylation. ADP-ribosylation of dsDNA, ssRNA and ssDNA were catalysed by PARP3, PARP10 cat and TRPT1, respectively.

ADP-ribosylation of proteins and DNA is reversible. We wanted to investigate whether RNA ADP-ribosylation by PARP10 is also a reversible process. Since PARP10 can ADP-ribosylate both 5′ and 3′ phosphorylated ends of RNA, we tested both of these modified oligos as substrates for well characterized human ADP-ribosylhydrolases: PARG, TARG1, MACROD1, MACROD2 and ARH1-3. We also tested human NUDT16 which is known to cleave the pyrophosphate bond in ADP-ribosylated proteins to generate phospho-ribose modified proteins (21). All above mentioned hydrolases, except for ARH1 and ARH2, were able to remove ADP-ribosylation from either 5′ or 3′ phosphates at the end of RNA substrates (Figure 2A and B). Importantly, catalytically inactive mutants of MACROD1 (G270E) and ARH3 (D77A) (27,30) did not remove ADPr from phosphorylated-ssRNA (Figure 2C), demonstrating that the enzymatic activity of these ADP-ribosylhydrolases is required for efficient removal of ADP-ribose from RNA phosphorylated ends. Together, these results demonstrate that ADP-ribosylation of phosphorylated-RNA oligos catalysed by PARP10 is a reversible process.

Previous studies have shown PARP10 to be an interferon induced gene that inhibits replication of Venezuelan equine encephalitis virus (VEEV) and other alphaviruses (42), yet the physiological substrates for PARP10 antiviral activity remain unknown. Thus, we tested viral macrodomain-containing hydrolases from VEEV and severe acute respiratory syndrome coronavirus (SARS CoV) for their ADP-ribosylhydrolase activity on RNA substrates. These viral hydrolases are known to support the ability of viruses to replicate in host cells (38,39,43–45), but their physiological substrates have yet to be identified. Strikingly, 5′ and 3′ phosphorylated ssRNA ADP-ribosylated by PARP10 could be efficiently reversed by the addition of viral macrodomain proteins (Figure 2D). The ability of viral macrodomains to remove ADPr from PARP10-modified phosphorylated-ssRNA could indicate a potential biological role for PARP10 in antiviral response acting on viral RNAs and the role of viral macrodomains in suppressing this function. Viral macrodomains could also reverse ADP-ribosylation of both double stranded and single stranded DNA similar to single stranded RNA modification (Figure 2E).

### NAD^+^ dependent phosphotransferase TRPT1 reversibly caps RNA ends

Next we decided to check several other human PARPs for their ability to modify RNA. We tested full length PARP3 and PARP16 (46); catalytic domains of PARP4, Tankyrase2 (47), PARP10, PARP11, PARP12, PARP13, PARP14 and PARP15 and a highly diverged PARP-like protein sometimes annotated as 18th human PARP–TRPT1 (4,48) for RNA ADP-ribosylation activity.

**Figure 3.**
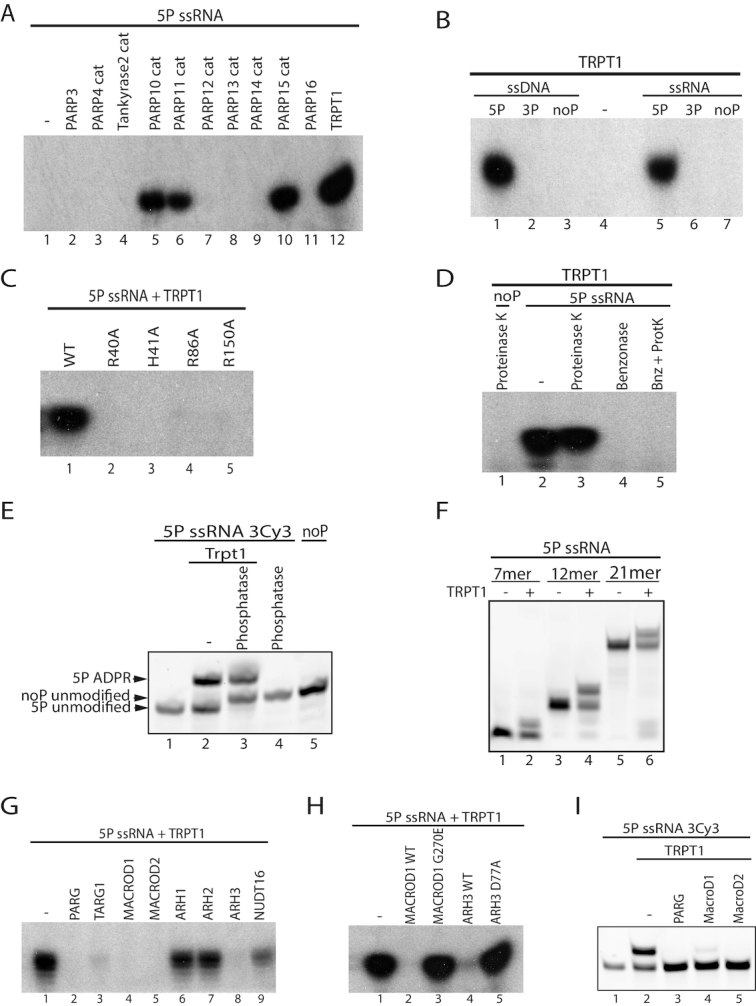
RNA and DNA ADP-ribosylation catalysed by TRPT1. (**A**) ADP-ribosylation of 5′ phosphorylated RNA end by several members of PARP superfamily. (**B**) ADP-ribosylation of ssDNA and ssRNA with or without phosphate at 5′ or 3′ end. TRPT1 specifically modifies 5′ phosphorylated oligo ends. (**C**) The Arg-His-Arg-Arg tetrad is essential for ADP-ribosylation of RNA by TRPT1. Point mutation of any tetrad amino acid residue to alanine leads to complete loss of TRPT1 activity. (**D**) TRPT1 mediated RNA ADP-ribosylation is further treated with: Proteinase K & SDS, Benzonase alone or Benzonase followed by Proteinase K treatment (Bnz + ProtK). (**E**) ssRNA with phosphate group at 5′ end and labelled with Cyanine3 tag at 3′ end (5P ssRNA 3Cy3) modified in the presence of TRPT1 and further treated with CIP. To visualize Cyanine3 label fluorescence gels were imaged using PharosFX imager. (**F**) TRPT1 mediated modification of different length of RNA oligos (7, 12 and 21mer length). (**G**) RNA ADP-ribosylation by TRPT1 can be reversed by human ADP-ribosylhydrolases – PARG, TARG1, MACROD1, MACROD2 and ARH3. (**H**) Enzymatically dead mutants of MACROD1 and ARH3 are unable to reverse RNA ADP-ribosylation by TRPT1. (**I**) RNA ADP-ribosylation of ssRNA with 5P ssRNA 3Cy3 catalysed by TRPT1 reversed by PARG, MACROD1 and MACROD2. Gels were visualized using Cyanine3 label and PharosFX imager.

We observed that, in addition to PARP10, PARP11, PARP15 and human TRPT1 were also able to ADP-ribosylate 5′ phosphorylated ssRNA (Figure 3A, lanes 5, 6, 10 and 12). However, in the conditions tested, the other PARPs were unable to ADP-ribosylate 5′phosphorylated ssRNA (Figure 3A). We focused on the ADP-ribosylation activity of TRPT1. We wanted to test whether the ADP-ribosylation by TRPT1 was phosphate dependent and had specificity towards DNA and/or RNA. For this, we tested ssDNA and ssRNA oligo with a phosphate group at either 5′ or 3′ end or without phosphate group (noP). We observed TRPT1 can ADP-ribosylate both DNA and RNA but only in the presence of 5′ phosphorylated end (Figure 3B). We observe TRPT1 based RNA modification is time and NAD^+^ concentration dependent (Supplementary Figure S2A and B) however independent of MgCl_2_ (Supplementary Figure S1C, lanes 3 and 4). Conserved amino acid residues Arg-His-Arg-Arg are essential for 2′ phosphotransferase function of yeast Tpt1 (34,49). Based on sequence alignment we mutated the corresponding residues in human TRPT1 into single alanine based point mutations (R40A, H41A, R86A and R150A). These point mutants were unable to ADP-ribosylate 5′ phosphorylated end of RNA (Figure 3C). This suggests ADP-ribosylation at 5′ end of RNA is also mediated via the same active site as originally studied for NAD-dependent 2′ RNA phosphotransferase activity of yeast Tpt1. To further establish that the ADP-ribosylation signal observed in the presence of TRPT1 was an RNA dependent modification, we further treated the reaction with Proteinase K or Benzonase. The band observed in presence of TRPT1 and 5′P ssRNA was resistant towards Proteinase K treatment but not Benzonase, which validates the band to be nucleic acid related ADP-ribosylation (Figure 3D). We treated 5P ssRNA Cyanine3 tagged oligo (5P ssRNA 3Cy3) with TRPT1, which generated 50% of the ADP-ribosylated product of slower mobility in our conditions (Figure 3E, lanes 1 and 2). Treatment of unmodified 5′P oligo with CIP led to a band that migrated slower, similar to the non-phosphorylated oligo (Figure 3E, lane 1 versus lanes 4 and 5). CIP treatment of TRPT1 catalysed RNA substrate leaves the upward shifted ADP-ribosylated RNA oligo intact but the lower unmodified RNA oligo migrates similar to non-phosphorylated oligo (Figure 3E, lanes 3 and 5). These results confirm ADP-ribosylation mediated by TRPT1 is on the RNA oligo and the modification occurs on the phosphate group at the 5′ end which protects RNA against further dephosphorylation by CIP. We also tested smaller 7mer and 12mer RNA oligos and established that TRPT1 activity is not affected by the length of RNA oligo (Figure 3F).

Similar to PARP10 mediated RNA ADP-ribosylation, we wanted to test if the TRPT1 catalysed RNA modification could be reversed by known human ADP-ribosylhydrolases. We observe the removal of ADPr signal by PARG, TARG1, MACROD1, MACROD2, ARH3 and NUDT16 (Figure 3G). ARH1 and ARH2 were unable to reverse the RNA modification. Catalytically inactive mutants of MACROD1 (G270E) and ARH3 (D77A) were inactive compared against the wild-type hydrolase as seen earlier for PARP10 (Figure 3H). TRPT1 mediated DNA modification can also be reversed by the above tested human hydrolases (Supplementary Figure S2C). We also tested hydrolase function on the ADP-ribosylated 5P ssRNA 3Cy3 oligo. The phosphorylated oligo when treated with TRPT1 produces a slow migrating band and a faster migrating unmodified RNA band (Figure 3I, lane 2). Further treatment with hydrolases: PARG, MACROD1 and MACROD2 reverses the modification observed by the loss of the slow migrating ADP-ribosylated band (Figure 3I, lane 3–5). However, the reversal of modification by the hydrolases does not change the migration pattern of the lower unmodified band (Figure 3I, lane 3–5), that still matches the migration pattern of phosphorylated ssRNA (as seen in Figure 3I, lane 1 versus 3–5). This confirms the hydrolase mediated hydrolytic cleavage of ADPr group does not affect the phosphate group on which the modification is covalently attached.

### ADP-ribosylation activity of TRPT1 is conserved in different species

**Figure 4.**
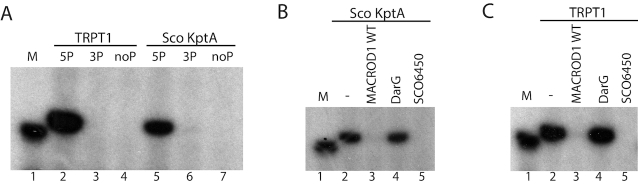
RNA ADP-ribosylation activity of TRPT1 is evolutionarily conserved. (**A**) KptA homolog from *Homo sapiens* (TRPT1) and bacterial *S. coelicolor* (Sco KptA) were tested with RNA oligo with or without phosphate group at 5′ or 3′ end of RNA and Cyanine3 label at the non-phosphorylated end. 5′ ^32^P RNA oligo with Cyanine3 at 3′ end was used as size marker in lane 1. (**B**) RNA ADP-ribosylation catalysed by Sco KptA can be reversed by human MACROD1 and *S. coelicolor* macrodomain containing protein Sco6450 but not by *Mtb* DarG. (**C**) *S. coelicolor* macrodomain containing protein Sco6450 can also reverse RNA ADP-ribosylation mediated by TRPT1.

TRPT1 homologues are distributed across eukaryal, archaeal and bacterial domains of life. In *E. coli* bacteria, these proteins are usually referred to as KptA. We wanted to test if RNA ADP-ribosylation by KptA homologs is conserved across different species. In addition to TRPT1, we also tested KptA homolog from *S. coelicolor* (Sco KptA/ SCO3953) with different RNA substrates. As with earlier for TRPT1 experiment, we observe that Sco KptA could also exclusively ADP-ribosylate RNA at 5′ phosphorylated end (Figure 4A, lanes 2 and 5). Since *S. coelicolor* also possesses a MacroD-like protein SCO6450 similar to human MACROD1 and MACROD2 we were interested to investigate if this macrodomain could function as potential hydrolase to remove ADP-ribosylation mediated by Sco KptA. Using MACROD1 as a positive control for removal of ADP-ribosylation mediated by both Sco KptA and TRPT1 we tested SCO6450 and another known bacterial macrodomain fold containing hydrolase DarG (25). We observed that SCO6450 was proficient at reversing RNA ADP-ribosylation mediated by both Sco KptA and TRPT1, however, DarG was inactive against KptA mediated modification (Figure 4B and C).

## DISCUSSION

ADP-ribosylation is an important chemical modification which helps cells to adapt and survive while maintaining their genomic integrity when faced with challenging environmental conditions. Classic macromolecular targets of ADP-ribosylation have been proteins, however, there have been several studies in the past few years that have demonstrated DNA as an important target for ADP-ribosylation (25–29). Here, we set out to uncover if any member of the ADP-ribosyltransferase family could also ADP-ribosylate RNA. We demonstrate, for the first time, that ADP-ribosylation of RNA can be catalysed by a few members of PARP family—PARP10, PARP11, PARP15 and a PARP-like protein –TRPT1 previously characterized as an NAD^+^ dependent phosphotransferase (50,51).

PARP10 was one of the first intracellular mono(ADP-ribosyl)ating ARTs identified (52). In addition to the catalytic domain, PARP10 also contains a RNA recognition motif (RRM), two functional ubiquitin interaction motifs (UIM), a sequence that promotes nuclear targeting as well as nuclear export and a motif that mediates interaction with PCNA (PIP) (40,52–55). While several protein targets of PARP10 have been suggested (35), the physiological role of PARP10 is unclear. Our study sheds further light on the potential biological function of PARP10 through modification of RNA. Our results show PARP10 can ADP-ribosylate phosphorylated RNA ends with a modest preference for 5′ over 3′ ends. PARP10 mediated RNA ADP-ribosylation is resistant to phosphatase treatment which would indicate a novel RNA capping mechanism possibly protecting the RNA against the nuclease attack. While the biological relevance for this RNA based modification is currently unknown we postulate a potential role in the innate immune response. PARP10 has previously been shown to be induced by interferon and can inhibit viral replication (42,43,56). PARP10 can also inhibit the activation of NF-κB which is activated during infection (55). ADP-ribosylation of RNA by PARP10 could act as a signal/marker to initiate an appropriate immune response. PARP10 has an inhibitory effect on alphavirus replication and on protein biosynthesis (42,56). These inhibitory effects could potentially be mediated via RNA ADP-ribosylation, where the ADPr moiety acts as a RNA cap thereby preventing RNA translation or triggering signal transduction. The presence of RRM domain in PARP10 could have a role in differentiating foreign RNA of invading pathogens from host RNA to work in tandem with the catalytic domain to ADP-ribosylate RNA and to further initiate the immune response. Similar function has been observed for the apoptotic role of PARP10 whereby the RRM domain contributed to pro-apoptotic activity together with the catalytic domain (57). While RNA ADP-ribosylation could provide an interesting link towards explaining the anti-viral role of PARP10, equally this activity could function in initiating or inhibiting translation thus effecting a cascade of signal transduction.

Several human ADP-ribosyl hydrolases PARG, TARG1, MACROD1/2 and ARH3 can reverse RNA ADP-ribosylation mediated by PARP10. Localization of these hydrolases to nucleus, cytoplasm and mitochondria (17,30,58,59) suggests that RNA ADP-ribosylation is utilized in different cellular compartments. Interestingly, in addition to human hydrolases we also observe that VEEV and SARS viral macrodomain-containing hydrolases can remove RNA ADP-ribosylation mediated by PARP10. This ability of viral macrodomains could indicate a mechanism of pathogenesis by counteracting antiviral activity of PARPs. This could make viral macrodomains good candidates as a potential drug target to combat pathogenesis.

In addition to PARP10, PARP11 and PARP15 we also show ADP-ribosylation of RNA catalysed by TRPT1, an ancestral relative of PARP superfamily which is sometimes referred to as the 18^th^ member of PARP family. This gene is highly conserved in eukaryotic, archaeal and bacterial domains of life. While the human gene is known as TRPT1, the yeast and bacterial version of TRPT1 are referred to as Tpt1 and KptA, respectively. The yeast homologue has been characterized for its role in tRNA splicing acting as a NAD^+^ dependent 2′-phosphotransferase (50,60). The enzymatic role of 2′-phosphate removal by Tpt1 occurs in a two chemical steps process – first, the 2′-phosphate reacts with NAD^+^ to release nicotinamide and form 2′-phospho-ADP-ribose RNA intermediate and second step involves generation of ADP-ribose 1″-2″ cyclic phosphate and leaving behind the RNA with hydroxyl group at 2′ end. While 2′-phosphotransferase activity is conserved across all diverse homologues of TRPT1 (31) there is no evidence of intron containing tRNA (that would need TRPT1 activity for splicing) and/or pathway which would generate RNA with 2′-phosphate in most of the organisms except in plants and fungal species (31,61). Furthermore, TRPT1 knockout cells from mouse exhibit levels of tRNA splicing comparable to the wild type cells (61). Although some bacteria possess introns in their tRNAs, they are self-splicing introns with very limited distribution to several representatives of proteobacteria and cyanobacteria (62). Therefore, the functional role of these widely conserved TRPT1 genes in other species remains elusive. A recent study has demonstrated that several archaeal species such as *Aeropyrum pernix*, Pyrococcus horikoshii and Archaeoglobus fulgidus and bacterial Clostridium thermocellum possess Tpt1 protein that can ADP-ribosylate RNA at 5′-phosphorylated ends (31). In our study, we show that TRPT1 from a higher eukaryote (human) and from a bacterium (*Streptomyces* species) can also ADP-ribosylate 5′-phosphorylated RNA—revealing that RNA ADP-ribosylation activity is widespread among Trpt1 proteins.

To summarize, our results identify RNA as a novel target of reversible ADP-ribosylation that can be catalysed by both PARP and TRPT1 classes of ARTs *in vitro*. This modification of RNA occurs on phosphorylated terminal ends of RNA; it can be made by PARP10 and TRPT1 ARTs and reversed by several known ADP-ribosylhydrolases. Efficient *in vitro* activities on RNA substrates by these enzymes suggest that RNA ADP-ribosylation reactions could be relevant *in vivo*. We hypothesize that TRPT1/PARP10 could potentially mediate ADP-ribosylation signalling on RNA substrates as an on/off switch thereby controlling the functional state of RNA, protecting RNA ends or act as a platform for recruiting other proteins. In addition we also demonstrate other PARPs—PARP11 and PARP15 to ADP-ribosylate phosphorylated RNA ends, however further characterization is required to reveal the functional role of these proteins.

## Supplementary Material

gkz305_Supplemental_File
