# Supplementary material for: Reversible ADP-ribosylation of RNA
Source: Nucleic Acids Res. 2019 Apr 26;47(11):5658–69. doi: 10.1093/nar/gkz305 (PMC6582358; doi:10.1093/nar/gkz305)
Supplement: gkz305_Supplemental_File [file gkz305_supplemental_file.pdf]

Supplementary Figure 1.

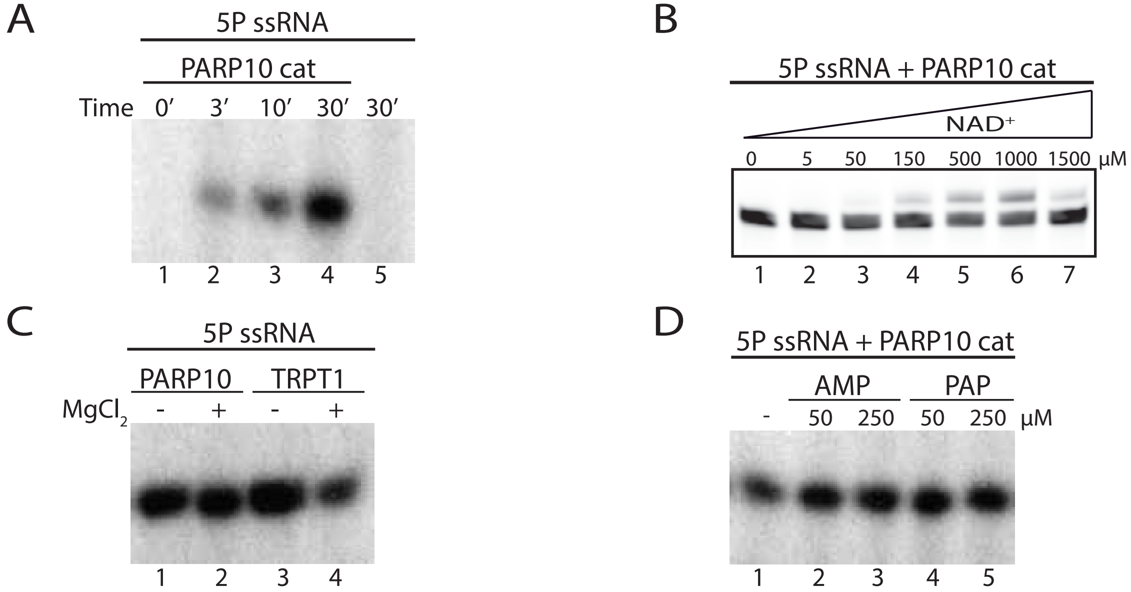

Supplementary Figure 1. ADP-ribosylation of 5' phosphorylated RNA end mediated by PARP10 cat is dependent on (A) time and (B) NAD^+ concentration. (C) ADP-ribosylation of 5' phosphorylated RNA end in presence or absence of MgCl2 in buffer. ADP-ribosylation is catalysed by PARP10 or TRPT1. (D) PARP10 cat mediated RNA ADP-ribosylation in presence of adenosine mono-phosphate (AMP) or 3'-phosphoadenosine 5'-phosphate (PAP).

Supplementary Figure 2.

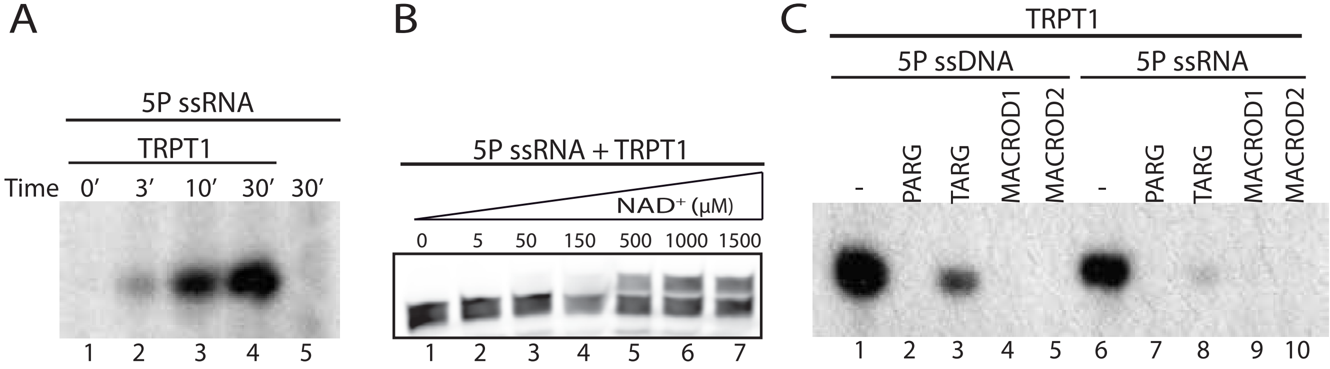

Supplementary Figure 2. ADP-ribosylation of 5' phosphorylated RNA end mediated by TRPT1 is dependent on (A) time and (B) NAD^+ concentration. (C) DNA and RNA ADP-ribosylation by TRPT1 can be reversed by known human hydrolase such as PARG, MACROD1 and MACROD2. TARG1 also weakly removes ADP-ribosylation.
